# Biomaterial used to counteract ridge reduction following the removal of adjacent teeth: A randomized controlled multicenter study

**DOI:** 10.1002/jper.70084

**Published:** 2026-03-23

**Authors:** Denis Cecchinato, Enrico Corrà, Eriberto Bressan, Marika Gervasi, Marco Menoncin, Enrico Savio, Marco Toia

**Affiliations:** ^1^ Institute Franci Private Practice Padova Italy; ^2^ Private Practice Poiana Maggiore Vicenza Italy; ^3^ Department of Neurosciences, School of Dentistry University of Padova Padova Italy; ^4^ Department of Industrial Engineering University of Padova Padova Italy; ^5^ Department of Oral and Maxillofacial Surgery and Oral Medicine Faculty of Odontology Malmö University Malmö Sweden

**Keywords:** alveolar ridge preservation, alveolar ridge resorption, bone substitutes, collagen membrane, multicenter study, randomized controlled trial, tooth extraction

## Abstract

**Background:**

Alveolar ridge resorption (ARP) is a well‐recognized consequence of tooth extraction, and multiple adjacent extractions may lead to greater ridge reduction than single‐tooth extractions. This multicenter randomized controlled trial aimed to assess ridge remodeling after removing 2 adjacent teeth and whether socket grafting with deproteinized bovine bone mineral with collagen (DBBM‐C) plus a collagen membrane could counteract the ridge reduction.

**Methods:**

Forty‐two patients requiring 2 adjacent tooth extractions were randomly assigned to either Test (DBBM‐C graft + collagen membrane) or Control (natural healing). Impressions were taken immediately after extraction and at 6 months, and models were analyzed to measure changes in horizontal ridge width (bucco‐lingual at 3 mm below the crest) and vertical ridge height (buccal and lingual).

**Results:**

At 6 months, grafted sites had significantly less ridge reduction than controls. Horizontal ridge width reduction averaged 57.7% in Control versus 23.0% in Test (*p* < 0.001). Vertical height loss was also greater in controls (buccal 3.0 mm vs. 1.5 mm; lingual 2.2 mm vs. 1.3 mm; *p* < 0.001). Both mesial and distal sockets benefited similarly from grafting, with comparable preservation at each site.

**Conclusion:**

Filling adjacent extraction sockets with DBBM‐C and covering with a collagen membrane significantly reduced horizontal and vertical alveolar ridge resorption compared to unassisted healing. This approach effectively preserves bone volume after multiple tooth extractions, which may facilitate later implant placement.

**Plain language summary:**

When 2 neighboring teeth are removed, the area tends to shrink in width and height as it heals. This bone loss can make it more difficult to place dental implants later. In this study, we tested a method to help maintain the bone after removing 2 adjacent teeth. In the Test group, the empty tooth sockets were filled with a bone graft material (deproteinized bovine bone mineral and collagen) and covered with a collagen membrane. In the Control group, the sites were left to heal physiologically. A total of 42 patients participated, each needing 2 side‐by‐side teeth removed. After 6 months of healing, we compared models of the patients from right after the extractions to those taken 6 months later. The results showed that the group with the bone graft and membrane had much less bone shrinkage than the group without them. The 2 extraction sites in the graft group maintained their bone height and width similarly. In summary, using a bone substitute material and membrane immediately after multiple tooth extractions helped preserve the jawbone, which could make future treatments like dental implants more successful.

## 1 INTRODUCTION

Findings from clinical studies as well as experiments using the dog model have documented that the alveolar ridge undergoes marked diminution after tooth extraction.^1^, ^2^, ^3^ The tissue reduction was reported to be more pronounced at the buccal/facial than at the palatal/lingual aspect of the ridge.^3^, ^4^, ^5^ To counteract this ridge reduction several alveolar ridge preservation (ARP) techniques were advocated.^6^, ^7^, ^8^, ^9^ In such ARP studies, various biomaterials were placed in a fresh extraction socket. It was observed that several studies have demonstrated that many grafting materials and ARP protocols can effectively influenced tissue diminution both in the short‐ and long‐term perspective.^10^, ^11^, ^12^, ^13^, ^14^, ^15^, ^16^, ^17^, ^18^

Furthermore, despite the efficacy reported for the ARP methods, the conclusions made in several publications were based on data targeting alterations occurring in the hard and soft tissue envelope of 1 single extraction sites. A few studies indicated that the removal of multiple adjacent teeth would cause proportionally more advanced tissue loss during healing than the removal of a single tooth.^19^, ^20^, ^21^, ^22^

The aim of the present clinical study was to determine (i) the degree of remodeling that occurs after multiple extractions of adjacent teeth, (ii) if socket grafting and wound protection with a barrier membrane could counteract ridge diminution.

## 2 MATERIALS AND METHODS

### 2.1 Study design

The study was designed as a prospective, randomized, controlled multicenter study and to comply with the Helsinki Declaration.

The clinical study was approved by the Ethical Committee of Veneto Region, “Azienda U.L.S.S. N.16”, Padova, Italy: N. 0044160, (approved January 8 2015) and was registered at the US National Institutes of Health (Clinicaltrials.gov): NCT02903667. The CONSORT 2010 statement was followed as a guideline when reporting the outcomes^23^ (Figure S1 in the online *Journal of Periodontology*). The study was conducted in 4 private clinics in Italy. At each clinic, an experienced clinician performed the surgical procedures and collected the data. A summary of patient distribution by center and group is reported in Table S1 in the online *Journal of Periodontology*.

### 2.2 Patient characteristics

Patients in need of implant restorations at 2 adjacent tooth sites in the canine/premolar or first molar regions of the mandible and incisor/canine/ premolar regions of the maxilla were included according to the following criteria: age >18 years; healthy (American Society of Anesthesiology classification 1 [ASA‐1]) or subjects with mild systemic disease (ASA‐2)^24^; willingness to comply with all study requirements and to sign informed consent; acceptable oral hygiene defined as full mouth scores ≤25%^25^; presence of intact extraction sockets following removal of the natural teeth or a marginal dehiscence defect of any of the facial bone walls of <3 mm. In addition, subjects were excluded if they presented with any of the following conditions: smoking more than ten cigarettes per day; history of radiotherapy in the head and neck region; uncontrolled diabetes; mucosal disease in the areas to be treated; situations that the principal investigator considered unsuitable for the surgical treatment (Table S2 in the online *Journal of Periodontology*).

The subjects were informed and asked to give their written informed consent. All patients received careful oral hygiene instructions and training in self‐performed plaque control. Patients were enrolled between April 2016 and December 2017, and all patients completed the 6‐month follow‐up by December 2018.

### 2.3 Interventions

After local anesthesia, full‐thickness buccal/facial and palatal/lingual flaps were elevated. Care was taken during the extraction of the 2 teeth to induce a minimum of trauma to the soft and hard tissue.

From an initial enrollment of 58 subjects, 16 did not meet the inclusion criteria after surgery due to the presence of extensive socket damage, including socket wall dehiscence of >3 mm and fracture of the interdental septum (Figure S1).

The 42 included subjects were randomly assigned into the 2 groups using a block randomization software immediately after the teeth extraction phase.

In each subject, the 2 adjacent extraction sites, were categorized as Socket 1 (mesial) and Socket 2 (distal).

**FIGURE 1:**
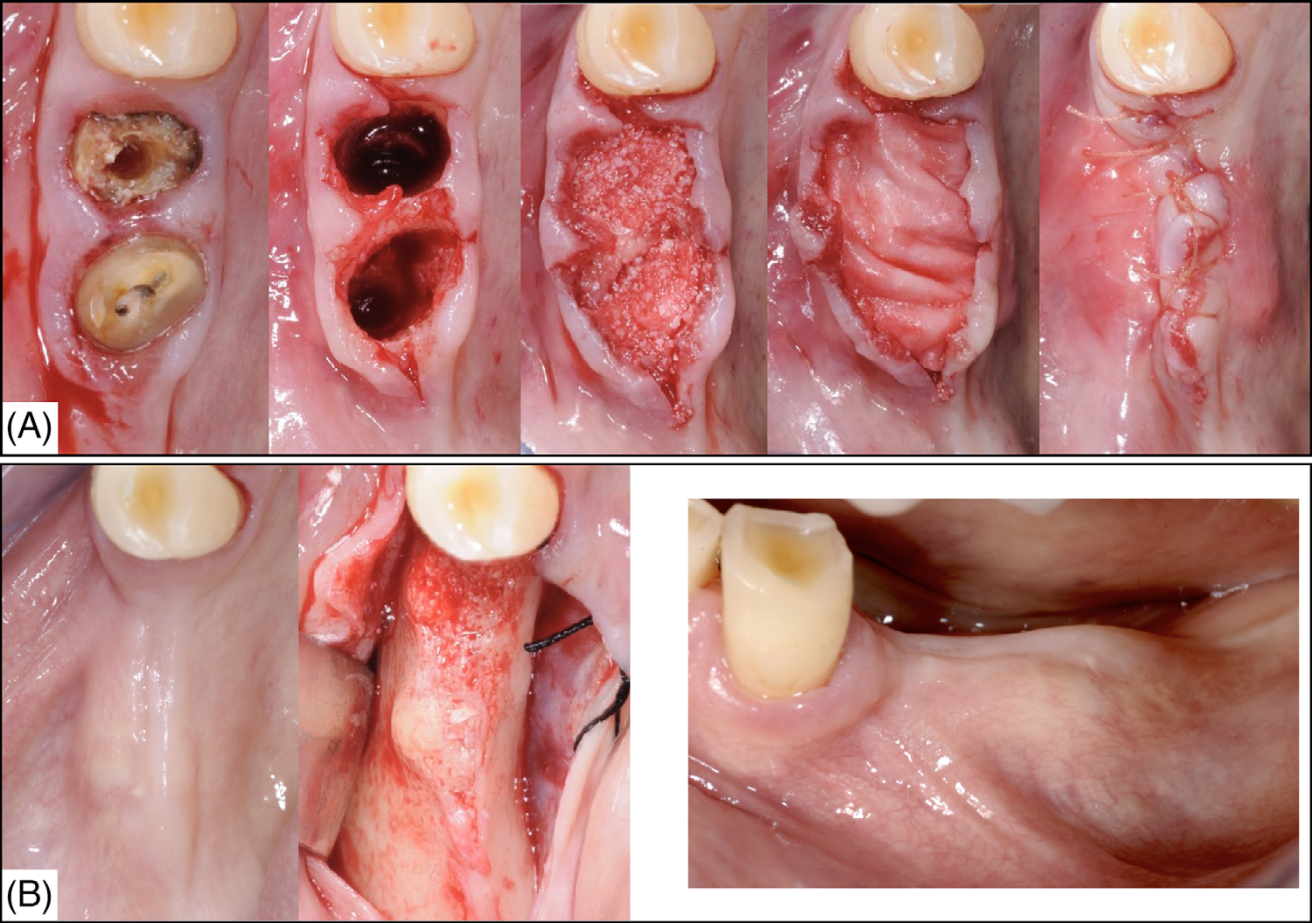
Clinical case from the Test group. Post‐extraction sites filled with DBBM‐C and covered with a resorbable membrane (A), and the 6‐month healing outcome (B).

**FIGURE 2:**
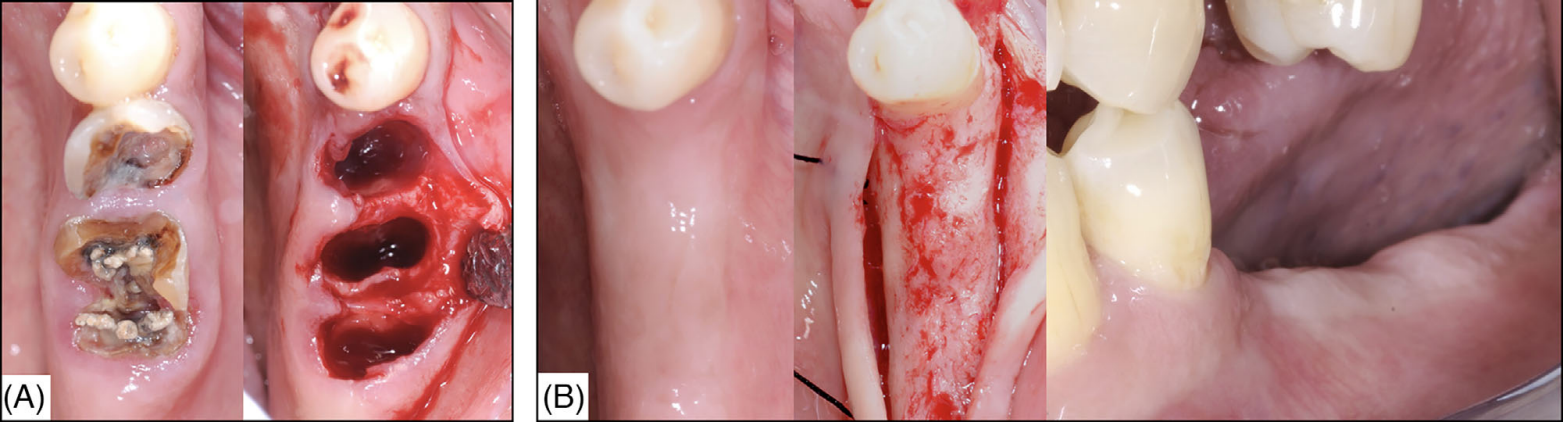
Clinical case from the control group. Post‐extraction sites (A) and the 6‐month healing outcome (B).

In the Test group, deproteinized bovine bone mineral with collagen* (DBBM‐C) was placed to fill the fresh extraction sockets, and the wound was covered with a resorbable membrane†.^26^ The full‐thickness flaps were advanced and sutured to achieve primary closure over the membrane (Figure 1). In the Control group, the walls of the fresh sockets were instrumented to promote bleeding and blood clot formation and allowed to undergo natural or physiological healing (Figure 2). As in the Test group, the flaps were replaced and secured by sutures.

An impression of the Test and Control sites was taken immediately after the end of the surgical procedure using alginate material (Hydrogum, Zhermack, Badia Polesine, Italy), and a stone model (Fujirock EP, GC Corporation, Tokyo, Japan) was produced.

All subjects were provided with a chlorhexidine (0.12%) ‐containing mouthrinse to be used 3 x/day for 2 weeks. Amoxicillin (2 gr) was administered to all patients 1 hour prior to surgery, and the antibiotic regimen was continued for 6 days after surgery, (1 gr twice a day).

After 6 months of healing, patients were recalled for the follow‐up visit, and new impressions were taken and a second set of stone models produced.

### 2.4 STL e‐models analysis

The stone models were scanned which enabled the polygonization of the acquired point with a sufficient mesh resolution. For each case, the *post‐ex e‐models* and the *6‐month e‐models* were imported and their respective superimpositions evaluated within the 3D analysis software (GOM Inspect 2018, GOM GmbH, Braunschweig, Germany).

Ridge alteration analyses was performed on STL e‐models, blinded, along the cross‐sections of the mesial and distal socket sites, and along the interdental septum.

**FIGURE 3:**
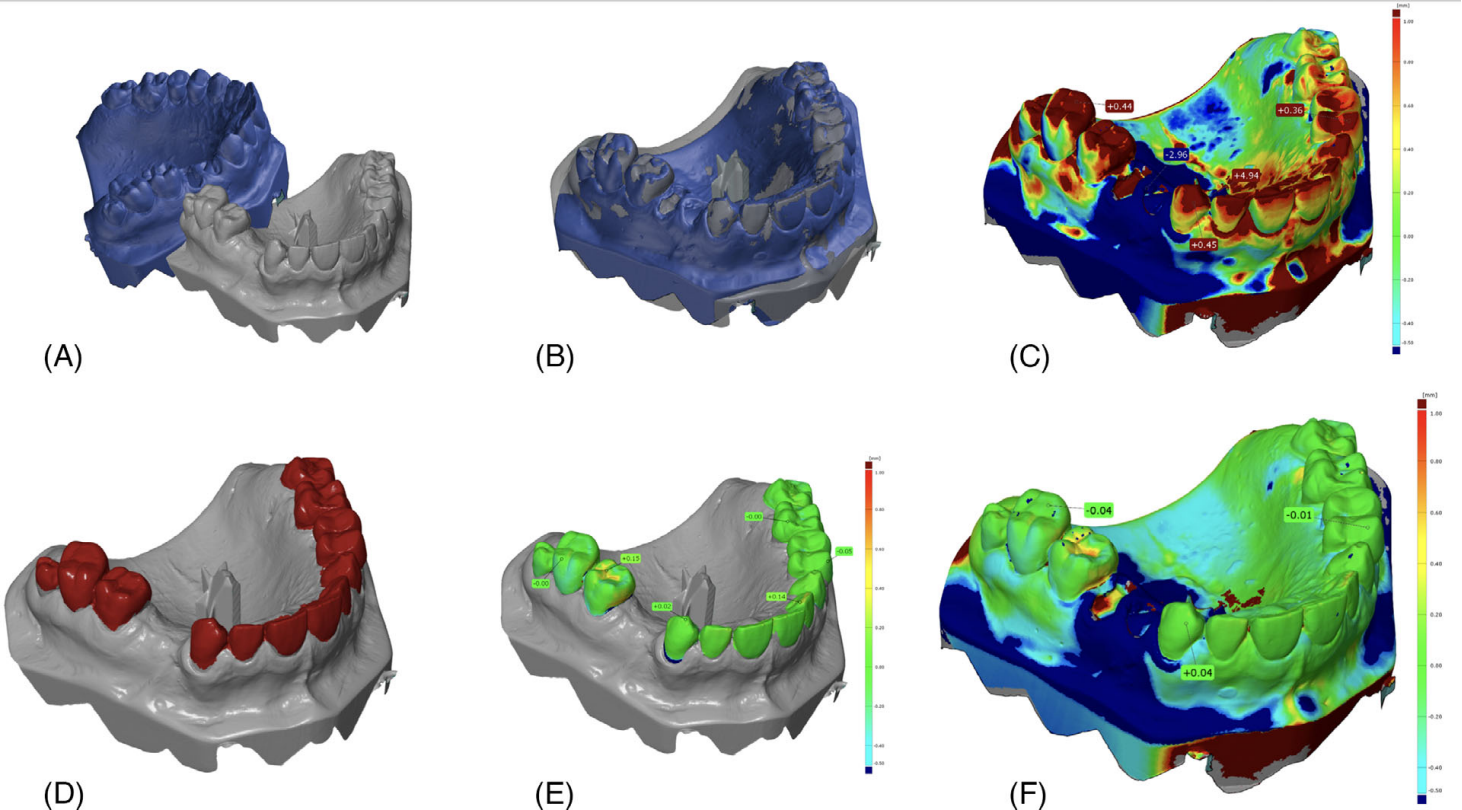
(A) Post‐ex e‐model and the 6‐month e‐model alignment. (B) First initial alignment. (C) Generated a deviation map using a 3‐point reference point system. (D,E) Best‐fit alignment using the remaining teeth, unaffected by surgery. (F) Validate the alignment procedure based on the tooth surface.

The cross‐sections were identified using the panoramic curve as reference parallel to the *z*‐axis of the model, which can be assumed as perpendicular to the occlusal plane^27^ (Figure 3). Each couple of models (*Post‐ex e‐model* and the *6‐month e‐model*) were superimposed using iterative alignment procedures (Figure 3A). A first initial alignment was imposed by a 3‐point reference points system to approach the 2 e‐models (Figure 3B) generating a deviation map not yet sufficiently accurate for further analysis (Figure 3C). Based on this pre‐alignment, a least squares local best‐fit alignment was imposed using the remaining teeth, unaffected by surgery (Figure 3D‐E). The surface selection used for the local best‐fit alignment was manually performed. The variability of the local best‐fit alignment (due to, e.g., selection area, operator) between the e‐models affects the dimensional measurements on the cross‐sections, because it affects the relative position between corresponding points. To consider the alignment dependency on the selected surface portion, the process of mesh selection and best‐fit alignment was iterated by 4 operators, twice for each e‐model. Changing the active alignment, the average deviations between *Post‐ex e‐model* and *6‐month e‐model* were calculated in the mesh area used for the alignment to validate the alignment procedure based on teeth surface (Figure 3F). The alignment reliability for subsequent dimensional evaluations reported an average variation of 0.02 (±0.03) mm. The standard deviation calculated for each average value was always lower than 0.5 mm and evaluated as clinically acceptable for the purpose of this study.^28^

**FIGURE 4:**
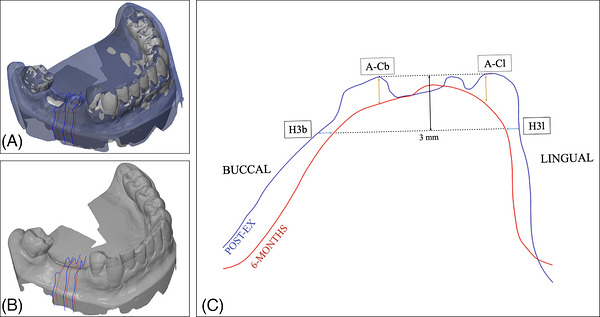
Cross‐section identification on aligned e‐models. Post‐ex e‐model is shown in blue (A), and the 6‐month e‐model in gray (B). Lines identified the cross‐sections in the sockets and in the interdental septum schematic representation of measurement specifications (C). A‐CB (vertical reduction at the buccal side); A‐CL (vertical reduction at the lingual side); H3b (horizontal reduction at 3 mm at the buccal side); H3l (horizontal reduction at 3 mm at the lingual side).

The cross‐sections identified in the *Post‐ex e‐model* were then fitted on the *6‐month e‐model* based on the active alignment (Figure 4), to allow measurements of local geometrical deviations according to International Organization for Standardization (ISO) 17450‐4:2017.^29^

The distances between corresponding points in the 2 e‐models define the dimensional variation of the ridge geometry during the 6‐month healing period. The analysis of each cross‐section of the Ridge (Socket 1, Socket 2, and Septum) involved the following measurements:

1. Bucco‐lingual (B‐L) “horizontal” (H) Ridge variation determined at 3 mm apical from the gingival peak: buccal (H3b) and lingual (H3l). The sum of the 2 measurements was reported as the total horizontal variation (H3).
2. Apico‐coronal (A‐C) “vertical” Ridge variation at buccal (B) and lingual (L) aspects, was performed using the gingival peak of the ridge as reference level as described in Figure 4.

This allowed the analysis of local geometrical deviations independently from any ridge deflections from the +*z* axis of the e‐models. The dimensional variation between the *6‐month e‐model* compared to the *Post‐ex e‐model*, was consistently registered as negative and denoted reduction.

The primary outcome of the trial was the horizontal ridge width reduction (B‐L reduction at 3 mm subcrestally). Secondary outcomes included vertical height changes (buccal and lingual).

### 2.5 Data analysis

Based on the detection of a difference of 3 mm in mean crest width difference between treatment groups, assuming a standard deviation of 3.0 mm^2^ and with an alpha error defined to 0.05 and beta error to 0.20 (power 80%), revealed that 17 subjects in each treatment group would be required. Considering for possible drop‐out a total of 50 subjects were included in the study.

Continuous variables were presented by means and standard deviations. Categorical and discrete variables were presented by frequency and percentage. The normal distribution was tested with the Shapiro–Wilk test. Since the distribution was not fulfilling the criteria for parametric testing, the Mann–Whitney U test was used to analyse each section separately using a specific software for data analysis (SPSS 21, IBM, USA). Moreover, the 2 post‐ex sites were also considered together and tested with Mixed linear model ‒ Estimation = Robust Maximum Likelihood (MLR) using Mplus (Mplus V.7 Muthén & Muthén, USA) in order to take into account within patient's effect.

The median absolute deviation (MAD) was used to detect the presence of univariate outliers. The significance level was set to .05.

## 3 RESULTS

Ridge reduction of the combined ridge changes calculated as the mean of Socket 1 (mesial), Socket 2 (distal), and the inter‐socket septum: (Socket 1 + Socket 2 + Septum)/2, representing the overall ridge unit.

### 3.1 B‐L (horizontal ridge reduction) at 3 mm

**FIGURE 5:**
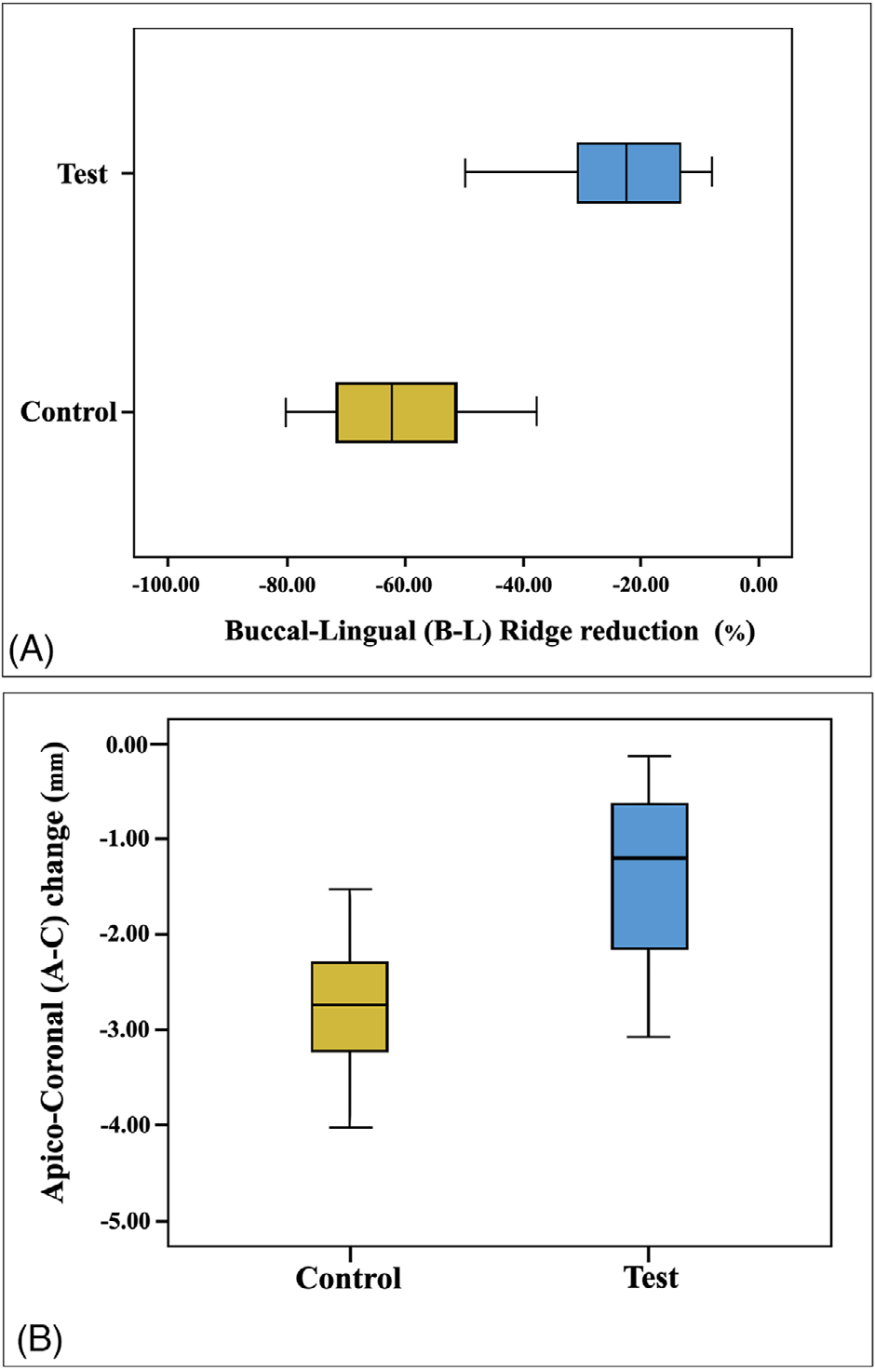
(A) Box‐plot representing the Buccal‐lingual Ridge reduction in the Control and Test groups. (B) Box plot representing the Apico‐coronal Ridge reduction in the Control and Test groups.

The mean B‐L reduction of the *6‐month e‐models* was 57.7 (±15.8) % and 23.0 (±12.1) % in the Control and in the Test group. The difference between the 2 groups was statistically significant (*p*‐value = 0.000) (Table 1) (Figure 5A).

**TABLE 1:** Ridge reduction of the 6‐month e‐models.

Ridge							
		Control			Test		
Parameter	*p‐*value	*n*	Mean (SD)	Median (min‐max)	*n*	Mean (SD)	Median (min‐max)
Bucco‐lingual (B‐L)							
Total (%)	0.000*	39	−57.7 (15.8)	−57.6 (−80.2; −26.2)	51	−23.0 (12.1)	−21.4 (−49.8; −3.9)
Total (mm)	0.000*	39	−6.4 (2.2)	−5.9 (−11.3; −2.3)	51	−2.7 (1.4)	−2.6 (−6.3; −0.4)
Lingual (L) (mm)	0.000*	39	−2.4 (1.0)	−2.2 (−5.5; −0.9)	51	−1.2 (0.6)	−1.1 (−3.3; −0.3)
Buccal (B) (mm)	0.000*	39	−4.1 (1.8)	−3.3 (−9.1; −1.2)	51	−1.5 (1.9)	−1.5 (−9.1; −1.2)
Apico‐coronal (A‐C)							
Total (mm)	0.000*	39	−2.6 (0.8)	−2.6 (−4.5; −0.8)	51	−1.4 (0.8)	−1.2 (−3.1; −0.1)
Lingual (L) (mm)	0.000*	39	−2.2 (0.6)	−2.2 (−4.0; −0.5)	51	−1.3 (0.8)	−1.0 (−3.7; −0.0)
Buccal (B) (mm)	0.000*	39	−3.0 (1.5)	−2.5 (−7.0; −1.0)	51	−1.5 (0.9)	−1.4 (−3.1; −0.1)

*Note*: Bucco‐lingual (B‐L) horizontal ridge variation at 3 mm apically from the gingival peak. Apico‐coronal (A‐C) vertical ridge variation.

* Mixed linear model ‒ Estimation = Robust Maximum Likelihood (MLR).

In both groups, the reduction was larger at the buccal than at the lingual aspect. The difference between the groups was not statistically significant. However, within the Control group, the buccal reduction was significantly greater than the lingual reduction (*p*‐value = 0.012).

### 3.2 A‐C ridge reduction

The mean total A‐C reduction was significantly greater in the Control group, −2.6 (±0.8) mm, than in the Testgroup, −1.4 (±0.8) (*p*‐value = 0.000).

The A‐CB reduction was −3.0 (±1.5) mm and −1.5 (±0.9) mm in the Control and in the Test group. Also, this difference between the 2 groups was statistically significant (*p*‐value = 0.000). Furthermore, also the A‐CL reduction was significantly greater in the Control, −2.2 (±0.6) mm, than in the Test group −1.3 (±0.8) mm (Test) (*p*‐value = 0.000) (Table 1, Figure 5B).

Individual dimensional changes for Socket 1 (1), Socket 2 (2), and the septum (3), reported separately:

In the Control group, the mean dimensional changes observed in Section 1 and Section 2 were comparable across all evaluated parameters. Specifically, the mean total horizontal reduction was −6.76 mm in Section 1 and −7.24 mm in Section 2 (difference = 0.48 mm; *p* = 0.564), while the total percentage reduction was −62.0% and −60.1%, respectively (difference = −1.9%; *p* = 0.725). Similarly, buccal (−4.12 vs. −4.79 mm; *p* = 0.393), lingual (−2.64 vs. −2.45 mm; *p* = 0.668), and A‐C dimensional changes did not differ significantly between the 2 sections (all *p* > 0.05). Overall, no statistically significant differences were detected between Section 1 and Section 2 within the Control group, indicating a comparable dimensional behavior of the 2 sites.

In the Test group, dimensional changes recorded in Section 1 and Section 2 were similar for all analyzed variables. The mean total horizontal reduction amounted to −2.59 mm in Section 1 and −3.18 mm in Section 2 (difference = 0.60 mm; *p* = 0.215), while the total percentage reduction was −21.4% and −26.5%, respectively (difference = 5.2%; *p* = 0.223). Buccal, lingual, and AC measurements also showed minimal differences between the 2 sections, with no statistically significant differences detected (all *p* > 0.05). These findings indicate that, within the Test group, Section 1 and Section 2 exhibited a comparable pattern of dimensional changes during healing. Detailed dimensional changes for each individual socket (Socket 1, Socket 2) and the inter‐socket septum are reported in the Table S3 in the online *Journal of Periodontology*.

## 4 DISCUSSION

The present clinical study demonstrated that the placement of DBBM‐C in 2 adjacent extraction sockets diminished the amount of ridge resorption that occurred in the non‐grafted control sites. It was furthermore documented that the amount of ridge “protection” was similar in each of the grafted adjacent sockets. It was also observed that the dimension of the septum between the extraction sockets was reduced during healing and that the septum change was apparently influenced by the grafting procedures.

The findings of the non‐grafted sites of the current study showed that the ridge reduction was similar in the combined sockets sites as in the individual mesial and distal sites.

The amount of ridge resorption, in the present study, is in general agreement with corresponding data reported in systematic review who followed the change that occurred at single tooth extraction sites.^30^, ^31^, ^32^

This observation is not in agreement with results presented by Lam^19^ who reported findings from 20 patients whose maxillary anterior teeth had been removed. Lam in 1960 concluded that, in such multiple extraction cases, the resulting ridge resorption was extensive. The present findings also differ from data reported by Al‐Shabeeb et al. and Al‐Askar et al. who, in animal experiments, observed that reduction of the crest was more pronounced at multiple than at single extraction sites.^21^, ^22^

In the grafted sites of the current study the ridge reduction was substantially smaller than in the non‐grafted sites. It was also observed that preservation of the ridge was similar in the individual mesial and distal socket sites. This finding agrees with Tomasi et al., who used the same methods, as in the present study, and reported that DBBM‐C placed in the single extraction site was effective in reducing ridge dimension.^33^

It should be emphasized that, in the present study protocol, socket sites with major dehiscence or septum defects were excluded. However, in most clinical cases, the degree of septal loss is unlikely to hinder implant placement, especially when implants are planned within the socket area. Nevertheless, the septum may be important in very narrow ridges or aesthetic zones, so future studies could specifically evaluate septal morphology. This implies that multiple sockets with major hard tissue defects, grafted or not, may not heal with the currently reported minimal reduction. It is proposed therefore that sites with pronounced hard and soft tissue defects should be included in further studies.

The horizontal reduction of the ridge in the grafted sites was significantly smaller than in the non‐grafted sites. The amount of ridge preservation following grafting was apparently similar at both the buccal and the lingual aspects.

On the contrary, in the non‐grafted sites, the buccal tissue loss was more pronounced than the corresponding lingual change. This observation agrees with findings from previous clinical studies where the healing of the entire ridge^33^ as well as of its bone compartment was investigated.^16^

In the non‐grafted group about 60% of the horizontal dimension, measured at 3 mm apically from the gingival peak, was lost. This degree of tissue reduction agrees with data reported in previous systematic reviews targeting single extraction sockets.^30^, ^31^, ^32^

It appears, therefore, that horizontal ridge resorption during healing in single and in multiple extraction sites undergo similar degree of change.

In the Control group, the height reduction of the multiple extraction sites amounted to 3.0 mm buccal and 2.2 mm at the lingual aspect. Similar amount of tissue loss occurred in the individual mesial and distal socket units. This amount of vertical change of the ridge is in agreement with results presented in systematic reviews ^11^, ^34^ and with finding by Araujo et al. ^16^ following single tooth extraction. Araújo et al. in 2015 used cone‐beam computed tomography (CBCT) scans to study bone wall alterations following single tooth extraction in humans. They observed that, already after 4 months, the vertical reduction of the buccal bone wall amounted to >4 mm, while the palatal bone wall was reduced about 2 mm.

At the grafted sites of the current study, the reduction of the vertical dimension was considerably smaller than in the non‐grafted sites. This agrees with conclusion from a randomized control clinical study in which it was evaluated the effect of the DBBM‐C in extraction sockets after removing the multiple teeth for an immediate complete denture.^35^ In the latter study, the authors, through the use of a CBCT scan, reported significantly less vertical and horizontal resorption in the grafted compared to the non‐grafted sites.

It should be pointed out, however, that the vertical height change, observed in this type of study may be closely related to the horizontal reduction.

In an animal study, Araujo and Lindhe in 2009^36^ observed that graft particles originally placed inside the hard tissue defect during healing become dislocated into the soft tissue. It should be recognized, therefore, that some of the influence of the grafting procedure used in the current study may have been attributed to the retention of the biomaterial in the soft tissue of the ridge.

These findings suggest that using DBBM‐C and a membrane when extracting 2 adjacent teeth can better preserve ridge dimensions. Clinicians should note, however, that even with grafting some resorption occurs; additional grafting at implant placement may still be needed in certain cases. Also, preserving ridge volume may allow implant placement to proceed without delay, but the timing should still consider complete healing (typically 4–6 months). Overall, patients receiving this ARP protocol can expect less ridge shrinkage, potentially simplifying future implant planning

This study has several limitations. First, the follow‐up period was limited to 6 months, which precludes any assessment of long‐term outcomes such as the stability of ridge dimensions or the fate of graft particles beyond the early healing phase. Second, volumetric changes were measured on dental stone casts rather than through direct radiographic imaging or histological evaluation; as a result, the observed dimensional changes reflect overall ridge contour remodeling (including both hard and soft tissue components) rather than purely bone‐specific alterations. Moreover, no histologic or biopsy data were collected, leaving the quality and turnover of the regenerated bone in grafted sites unknown. The healed ridge in grafted sockets is likely a composite of new bone and residual DBBM‐C particles, which may not be directly comparable to native bone in an ungrafted site and could have different implications for subsequent implant osseointegration. In addition, the study excluded extraction sockets with severe wall defects; while this criterion ensured more uniform baseline conditions, it limits the generalizability of the findings to more compromised sites with substantial bony deficiencies. Finally, all procedures were performed by experienced clinicians in specialized centers, which may have favorably influenced the outcomes and thus limits the external validity of the results to routine clinical practice.

## 5 CONCLUSIONS

(i) DBBM‐C associated with a collagen membrane, placed in adjacent extraction sites, reduced the overall ridge diminution including the horizontal and vertical components. (ii) The amount of ridge resorption was similar in the individual mesial and distal socket sites.

## AUTHOR CONTRIBUTIONS

**Dr. Denis Cecchinato**: Methodology; investigation; data curation; formal analysis; validation; visualization; writing—original draft; writing—review & editing; resources; supervision; project administration; funding Acquisition. **Dr. Enrico Corrà**: Methodology; software; data curation; investigation; validation; formal analysis; resources; writing—review & editing. **Prof. Eriberto Bressan**: Methodology; software; investigation; data curation; formal analysis; validation; visualization; writing—review & Editing. **Dr. Marika Gervasi**: Software; data curation; formal analysis; writing—original draft; writing—review & editing. **Dr. Marco Menoncin**: Methodology; software; data curation; formal analysis; validation; investigation; visualization; writing—original draft; writing—review & editing. **Prof. Enrico Savio**: Conceptualization; methodology; data curation; investigation; validation; formal analysis; supervision; resources; visualization; funding acquisition; writing review—editing. **Dr. Marco Toia**: Methodology; software; data curation; investigation; formal analysis; validation; visualization; writing—original draft; writing—review & editing; supervision. All authors have given final approval of the version to be published.

## CONFLICT OF INTEREST STATEMENT

The authors declare no conflicts of interest.

## CLINICAL TRIAL REGISTRATION

This study is part of a randomized controlled trial (RCT) registered with ClinicalTrials.gov (ID: NCT02903667). The title of the trial is “Biomaterial to Counteract Ridge Reduction Following the Removal of Multiple Adjacent Teeth.”

## Supporting information



Supporting information

Supporting information

Supporting information

Supporting information

Supporting information

## Footnotes

* Bio‐Oss Collagen, Geistlich Pharma, Wolhusen, Switzerland

† Bio‐Gide, Geistlich Pharma, Wolhusen, Switzerland
