# Supplementary figures and images for: Biomaterial used to counteract ridge reduction following the removal of adjacent teeth: A randomized controlled multicenter study
Source: J Periodontol. 2026 Mar 23;97(7):1481–90. doi: 10.1002/jper.70084 (PMC13380361; doi:10.1002/jper.70084)

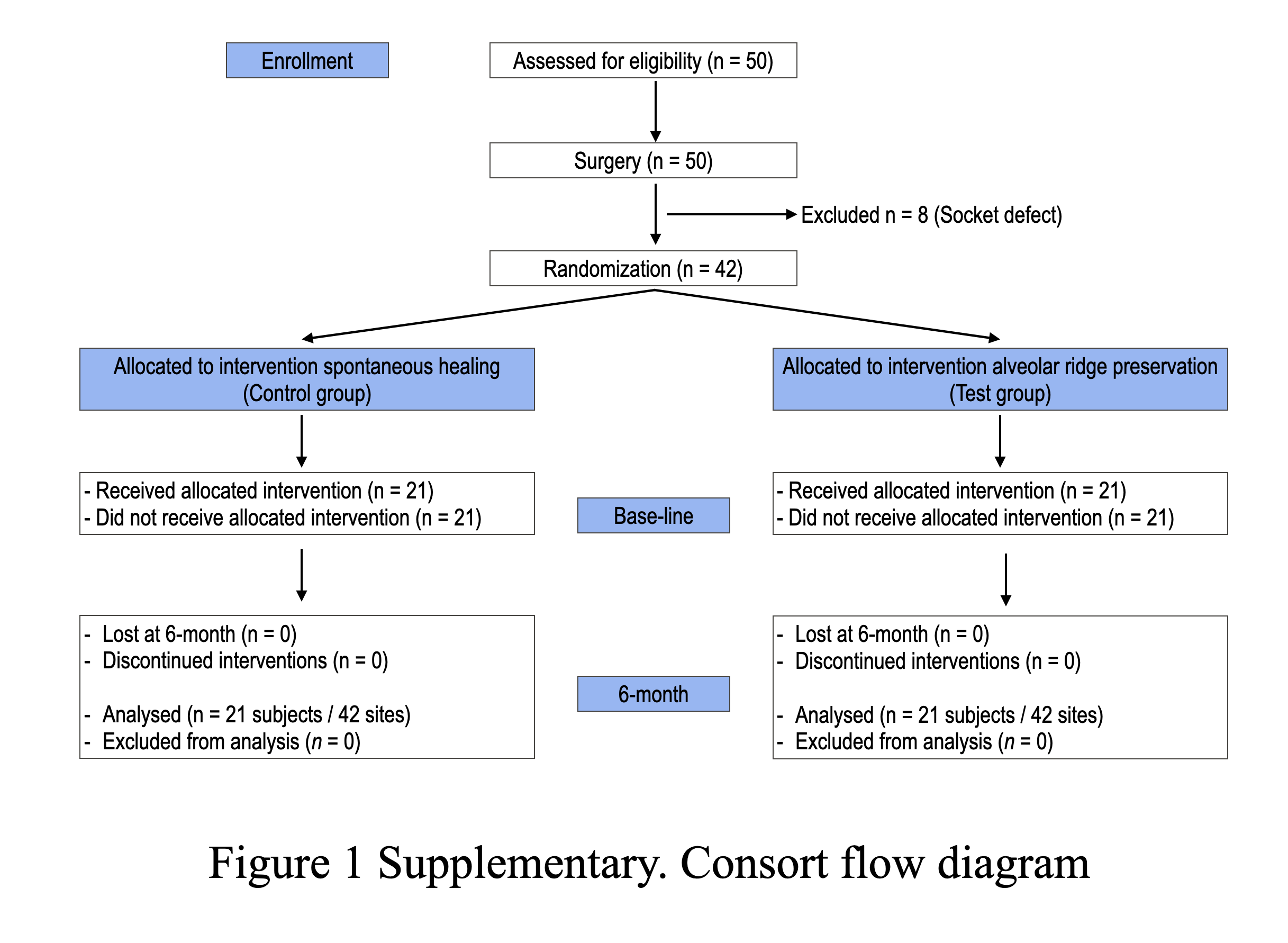

Supplement: Supplementary file 1 — Supporting information [file JPER-97-1481-s001.tiff]
