# Supplementary material for: Biomaterial used to counteract ridge reduction following the removal of adjacent teeth: A randomized controlled multicenter study
Source: J Periodontol. 2026 Mar 23;97(7):1481–90. doi: 10.1002/jper.70084 (PMC13380361; doi:10.1002/jper.70084)
Supplement: Supplementary file 2 — Supporting information [file JPER-97-1481-s004.docx]

**Table 1 Supplementary.** Forty-two patients were enrolled across four centers under a competitive recruitment model. This means the centers recruited patients at different rates, resulting in uneven patient counts per center. Twenty-one patients were assigned to the Test group (DBBM-C + collagen membrane) and twenty-one patients to the Control group (natural healing). The table below displays the allocation of patients by center, and the final row shows the total counts for each group.

| Center | Total Patients | Test Group (n) | Control Group (n) |
| --- | --- | --- | --- |
| Center EB | 10 | 5 | 5 |
| Center DC | 10 | 5 | 5 |
| Center EC | 11 | 6 | 5 |
| Center MT | 11 | 5 | 6 |
| Total | 42 | 21 | 21 |

Note: EB (Eriberto Bressan); DC (Denis Cecchinato); EC (Enrico Corrà); MT (Marco Toia).
