# Supplementary material for: Biomaterial used to counteract ridge reduction following the removal of adjacent teeth: A randomized controlled multicenter study
Source: J Periodontol. 2026 Mar 23;97(7):1481–90. doi: 10.1002/jper.70084 (PMC13380361; doi:10.1002/jper.70084)
Supplement: Supplementary file 3 — Supporting information [file JPER-97-1481-s002.docx]

| **Table 2 Supplementary.** Patient characteristics at baseline | | | | | | | | | | | | |
| --- | --- | --- | --- | --- | --- | --- | --- | --- | --- | --- | --- | --- |
|  |  | Control | | |  | Test | | |  | Total | | |
|  |  | n | % / mean (SD) | (min-max) |  | n | % / mean (SD) | (min-max) |  | n | % / mean (SD) | (min-max) |
| **Gender** | Female | 13 | 62% |  |  | 8 | 38% |  |  | 21 | 50% |  |
|  | Male | 8 | 38% |  |  | 13 | 62% |  |  | 21 | 50% |  |
|  |  |  |  |  |  |  |  |  |  |  |  |  |
| **Smoker** | No | 16 | 76% |  |  | 18 | 86% |  |  | 34 | 81 |  |
|  | Yes | 5 | 24% |  |  | 3 | 14% |  |  | 8 | 19 |  |
|  |  |  |  |  |  |  |  |  |  |  |  |  |
| **Age at surgery** |  | 21 | 66 (8.5) | (41-78) |  | 21 | 61.1 (10.3) | (39-83) |  | 42 | 63.5 (9.7) | (39-83) |
|  |  |  |  |  |  |  |  |  |  |  |  |  |
| **Socket location** |  |  | Socket 1 | Socket 2 |  |  | Socket 1 | Socket 2 |  |  |  |  |
|  | Maxilla | 1 | 12 | 13 |  | 1 | 13 | 14 |  |  |  |  |
|  |  | 4 | 14 | 15 |  | 4 | 14 | 15 |  |  |  |  |
|  |  | 5 | 24 | 25 |  | 2 | 22 | 23 |  |  |  |  |
|  |  |  |  |  |  | 1 | 23 | 24 |  |  |  |  |
|  |  |  |  |  |  | 3 | 24 | 25 |  |  |  |  |
|  |  | 10 |  |  |  | 11 |  |  |  | 21 | 50% |  |
|  |  |  |  |  |  |  |  |  |  |  |  |  |
|  | Mandible | 3 | 44 | 45 |  | 5 | 34 | 35 |  |  |  |  |
|  |  | 1 | 45 | 46 |  | 1 | 43 | 44 |  |  |  |  |
|  |  | 3 | 34 | 35 |  | 2 | 44 | 45 |  |  |  |  |
|  |  | 4 | 35 | 36 |  | 2 | 35 | 36 |  |  |  |  |
|  |  | 11 |  |  |  | 10 |  |  |  | 21 | 50% |  |
