# Supplementary material for: Biomaterial used to counteract ridge reduction following the removal of adjacent teeth: A randomized controlled multicenter study
Source: J Periodontol. 2026 Mar 23;97(7):1481–90. doi: 10.1002/jper.70084 (PMC13380361; doi:10.1002/jper.70084)
Supplement: Supplementary file 4 — Supporting information [file JPER-97-1481-s003.docx]

**Table 3 supplementary.** Individual dimensional changes for Socket 1 (1), Socket 2 (2), and the septum (3), reported separately.

|  | | | | | | | | | | | | | | | | | | | | | | | | | | | |
| --- | --- | --- | --- | --- | --- | --- | --- | --- | --- | --- | --- | --- | --- | --- | --- | --- | --- | --- | --- | --- | --- | --- | --- | --- | --- | --- | --- |
|  | | | | | | | | | | | | **95% Confidence Interval** | | | |  | | | | | | | | **Shapiro-Wilk** | | | |
|  | | **SECTION** | | **Ctrl - Test** | | **N** | | **Missing** | | **Mean** | | **Lower** | | **Upper** | | **Median** | | **SD** | | **Minimum** | | **Maximum** | | **W** | | **p** | |
| **Buccal-Lingual**  **(B-L)** |  |  |  |  |  |  |  |  |  |  |  |  |  |  |  |  |  |  |  |  |  |  |  |  |  |  |  |
| Total (mm) |  | 1 |  | Control |  | 13 |  | 0 |  | -6.758 |  | -7.923 |  | -5.593 |  | -6.380 |  | 1.9274 |  | -9.332 |  | -4.3517 |  | 0.866 |  | 0.047 |  |
|  |  |  |  | Test |  | 18 |  | 0 |  | -2.588 |  | -3.087 |  | -2.090 |  | -2.507 |  | 1.0017 |  | -4.391 |  | -1.0962 |  | 0.942 |  | 0.319 |  |
|  |  | 2 |  | Control |  | 11 |  | 0 |  | -7.242 |  | -8.646 |  | -5.838 |  | -6.931 |  | 2.0893 |  | -11.331 |  | -3.8898 |  | 0.979 |  | 0.962 |  |
|  |  |  |  | Test |  | 17 |  | 0 |  | -3.184 |  | -4.044 |  | -2.325 |  | -3.098 |  | 1.6717 |  | -6.320 |  | -0.8013 |  | 0.954 |  | 0.525 |  |
|  |  | 3 |  | Control |  | 15 |  | 0 |  | -5.551 |  | -6.783 |  | -4.318 |  | -4.872 |  | 2.2257 |  | -9.881 |  | -2.3020 |  | 0.937 |  | 0.348 |  |
|  |  |  |  | Test |  | 16 |  | 0 |  | -2.451 |  | -3.156 |  | -1.747 |  | -2.375 |  | 1.3223 |  | -4.450 |  | -0.4187 |  | 0.942 |  | 0.372 |  |
| Total (%) |  | 1 |  | Control |  | 13 |  | 0 |  | -62.045 |  | -70.935 |  | -53.155 |  | -69.315 |  | 14.7113 |  | -79.619 |  | -37.5811 |  | 0.886 |  | 0.086 |  |
|  |  |  |  | Test |  | 18 |  | 0 |  | -21.372 |  | -25.831 |  | -16.913 |  | -21.904 |  | 8.9670 |  | -39.348 |  | -8.1566 |  | 0.950 |  | 0.428 |  |
|  |  | 2 |  | Control |  | 11 |  | 0 |  | -60.102 |  | -68.177 |  | -52.027 |  | -62.294 |  | 12.0198 |  | -80.249 |  | -43.0405 |  | 0.955 |  | 0.705 |  |
|  |  |  |  | Test |  | 17 |  | 0 |  | -26.547 |  | -34.097 |  | -18.997 |  | -25.960 |  | 14.6850 |  | -49.803 |  | -7.8748 |  | 0.903 |  | 0.076 |  |
|  |  | 3 |  | Control |  | 15 |  | 0 |  | -49.599 |  | -59.201 |  | -39.997 |  | -42.056 |  | 17.3386 |  | -80.205 |  | -26.2187 |  | 0.899 |  | 0.093 |  |
|  |  |  |  | Test |  | 16 |  | 0 |  | -20.967 |  | -27.306 |  | -14.628 |  | -18.530 |  | 11.8962 |  | -43.162 |  | -3.8737 |  | 0.941 |  | 0.360 |  |
| Buccal (B) (mm) |  | 1 |  | Control |  | 13 |  | 0 |  | -4.117 |  | -5.073 |  | -3.162 |  | -3.726 |  | 1.5811 |  | -6.430 |  | -1.6117 |  | 0.931 |  | 0.348 |  |
|  |  |  |  | Test |  | 18 |  | 0 |  | -1.357 |  | -1.706 |  | -1.008 |  | -1.397 |  | 0.7022 |  | -2.959 |  | -0.1687 |  | 0.919 |  | 0.123 |  |
|  |  | 2 |  | Control |  | 11 |  | 0 |  | -4.795 |  | -6.220 |  | -3.370 |  | -4.295 |  | 2.1212 |  | -9.095 |  | -2.0888 |  | 0.924 |  | 0.354 |  |
|  |  |  |  | Test |  | 17 |  | 0 |  | -1.918 |  | -2.512 |  | -1.325 |  | -1.897 |  | 1.1538 |  | -3.660 |  | -0.1687 |  | 0.933 |  | 0.245 |  |
|  |  | 3 |  | Control |  | 15 |  | 0 |  | -3.498 |  | -4.460 |  | -2.537 |  | -2.727 |  | 1.7360 |  | -6.160 |  | -1.2400 |  | 0.818 |  | 0.006 |  |
|  |  |  |  | Test |  | 16 |  | 0 |  | -1.284 |  | -1.772 |  | -0.797 |  | -1.318 |  | 0.9147 |  | -2.665 |  | -0.1687 |  | 0.899 |  | 0.076 |  |
|  |  |  |  |  |  |  |  |  |  |  |  |  |  |  |  |  |  |  |  |  |  |  |  |  |  |  |  |
| Lingual (L) (mm) |  | 1 |  | Control |  | 13 |  | 0 |  | -2.641 |  | -3.227 |  | -2.055 |  | -2.703 |  | 0.9697 |  | -5.440 |  | -1.4100 |  | 0.765 |  | 0.003 |  |
|  |  |  |  | Test |  | 18 |  | 0 |  | -1.232 |  | -1.471 |  | -0.992 |  | -1.167 |  | 0.4821 |  | -1.875 |  | -0.5047 |  | 0.905 |  | 0.071 |  |
|  |  | 2 |  | Control |  | 11 |  | 0 |  | -2.447 |  | -3.237 |  | -1.657 |  | -2.355 |  | 1.1757 |  | -5.485 |  | -1.1438 |  | 0.832 |  | 0.025 |  |
|  |  |  |  | Test |  | 17 |  | 0 |  | -1.266 |  | -1.630 |  | -0.902 |  | -1.038 |  | 0.7079 |  | -3.333 |  | -0.4500 |  | 0.826 |  | 0.005 |  |
|  |  | 3 |  | Control |  | 15 |  | 0 |  | -2.052 |  | -2.435 |  | -1.669 |  | -2.113 |  | 0.6915 |  | -3.721 |  | -0.8911 |  | 0.935 |  | 0.327 |  |
|  |  |  |  | Test |  | 16 |  | 0 |  | -1.167 |  | -1.467 |  | -0.867 |  | -1.040 |  | 0.5633 |  | -2.015 |  | -0.2500 |  | 0.941 |  | 0.368 |  |
| **Apico-Coronal**  **(A-C)** |  |  |  |  |  |  |  |  |  |  |  |  |  |  |  |  |  |  |  |  |  |  |  |  |  |  |  |
| Buccal (B) (mm) |  | 1 |  | Control |  | 13 |  | 0 |  | -3.106 |  | -3.998 |  | -2.214 |  | -2.388 |  | 1.4758 |  | -6.933 |  | -1.6150 |  | 0.846 |  | 0.025 |  |
|  |  |  |  | Test |  | 18 |  | 0 |  | -1.596 |  | -2.031 |  | -1.160 |  | -1.781 |  | 0.8753 |  | -2.943 |  | -0.1425 |  | 0.954 |  | 0.486 |  |
|  |  | 2 |  | Control |  | 11 |  | 0 |  | -3.213 |  | -4.099 |  | -2.327 |  | -3.114 |  | 1.3190 |  | -5.738 |  | -1.0863 |  | 0.973 |  | 0.917 |  |
|  |  |  |  | Test |  | 17 |  | 0 |  | -1.352 |  | -1.865 |  | -0.840 |  | -1.279 |  | 0.9963 |  | -2.537 |  | -0.0850 |  | 0.830 |  | 0.005 |  |
|  |  | 3 |  | Control |  | 15 |  | 0 |  | -2.815 |  | -3.745 |  | -1.886 |  | -2.223 |  | 1.6784 |  | -7.025 |  | -1.0037 |  | 0.863 |  | 0.026 |  |
|  |  |  |  | Test |  | 16 |  | 0 |  | -1.602 |  | -2.068 |  | -1.136 |  | -1.361 |  | 0.8743 |  | -3.070 |  | -0.4662 |  | 0.862 |  | 0.021 |  |
|  |  |  |  |  |  |  |  |  |  |  |  |  |  |  |  |  |  |  |  |  |  |  |  |  |  |  |  |
| Lingual (L) (mm |  | 1 |  | Control |  | 13 |  | 0 |  | -2.263 |  | -2.740 |  | -1.786 |  | -2.155 |  | 0.7898 |  | -3.975 |  | -1.1283 |  | 0.959 |  | 0.736 |  |
|  |  |  |  | Test |  | 18 |  | 0 |  | -1.221 |  | -1.605 |  | -0.837 |  | -1.040 |  | 0.7727 |  | -3.024 |  | -0.1537 |  | 0.940 |  | 0.295 |  |
|  |  | 2 |  | Control |  | 11 |  | 0 |  | -2.295 |  | -2.620 |  | -1.970 |  | -2.224 |  | 0.4835 |  | -3.190 |  | -1.7371 |  | 0.932 |  | 0.433 |  |
|  |  |  |  | Test |  | 17 |  | 0 |  | -1.329 |  | -1.874 |  | -0.784 |  | -1.028 |  | 1.0595 |  | -3.733 |  | -0.0425 |  | 0.921 |  | 0.151 |  |
|  |  | 3 |  | Control |  | 15 |  | 0 |  | -2.134 |  | -2.483 |  | -1.785 |  | -2.334 |  | 0.6306 |  | -2.752 |  | -0.5225 |  | 0.850 |  | 0.017 |  |
|  |  |  |  | Test |  | 16 |  | 0 |  | -1.248 |  | -1.529 |  | -0.967 |  | -1.052 |  | 0.5272 |  | -2.239 |  | -0.6899 |  | 0.847 |  | 0.012 |  |
| (Total (mm) |  | 1 |  | Control |  | 13 |  | 0 |  | -2.684 |  | -3.090 |  | -2.279 |  | -2.627 |  | 0.6711 |  | -4.031 |  | -1.5542 |  | 0.976 |  | 0.957 |  |
|  |  |  |  | Test |  | 18 |  | 0 |  | -1.408 |  | -1.798 |  | -1.019 |  | -1.293 |  | 0.7837 |  | -2.754 |  | -0.2162 |  | 0.952 |  | 0.455 |  |
|  |  | 2 |  | Control |  | 11 |  | 0 |  | -2.754 |  | -3.220 |  | -2.288 |  | -2.831 |  | 0.6936 |  | -3.917 |  | -1.5238 |  | 0.992 |  | 0.999 |  |
|  |  |  |  | Test |  | 17 |  | 0 |  | -1.341 |  | -1.839 |  | -0.843 |  | -0.980 |  | 0.9682 |  | -3.066 |  | -0.1150 |  | 0.905 |  | 0.083 |  |
|  |  | 3 |  | Control |  | 15 |  | 0 |  | -2.475 |  | -3.035 |  | -1.914 |  | -2.358 |  | 1.0123 |  | -4.524 |  | -0.7631 |  | 0.964 |  | 0.754 |  |
|  |  |  |  | Test |  | 16 |  | 0 |  | -1.425 |  | -1.762 |  | -1.088 |  | -1.204 |  | 0.6330 |  | -2.539 |  | -0.5944 |  | 0.885 |  | 0.047 |  |
| Nota. The CI of the mean assumes sample means follow a t-distribution with N - 1 degrees of freedom | | | | | | | | | | | | | | | | | | | | | | | | | | | |
|  | | | | | | | | | | | | | | | | | | | | | | | | | | | |

# 
